# Spatiotemporal dynamics of Brucella immune evasion across infection stages

**DOI:** 10.3389/fimmu.2025.1707297

**Published:** 2026-01-08

**Authors:** Yu Su, Lihui Zhang, Ruihua Liu, Rina Sa, Lanbing Zheng

**Affiliations:** Scientific Research Department, Inner Mongolia Fourth Hospital (Chest Hospital), Hohhot, China

**Keywords:** *Brucella* spp., chronic infection, immune evasion, infection stages, spatiotemporal dynamics

## Abstract

*Brucella*, a major zoonotic pathogen, poses a significant threat to global public health and causes substantial economic losses in the livestock industry. It employs diverse and sophisticated immune evasion strategies to circumvent host surveillance, establishing and maintaining chronic infections that are difficult to treat and prone to relapse. While previous reviews have catalogued individual virulence factors—such as the VirB type IV secretion system—and their actions on pathways like TLR4 signaling, most analyses focus on isolated stages or mechanisms, overlooking the integrated, dynamic regulation spanning the entire infection course. A systematic framework explaining how Brucella modulates host immunity through multi-stage, multidimensional evasion is still lacking. This review synthesizes research from the past decade to delineate the Brucella immune-evasion network across four distinct stages: colonization, latency, acute disease, and chronic persistence. We propose a Spatiotemporal Dynamic Immune Evasion Model that unifies these processes, offering novel insights into the immunological basis of chronic brucellosis and providing a foundation for developing stage-specific therapeutics and next-generation vaccines with strong translational potential.

## 1 Introduction

Brucellosis is a significant zoonotic disease caused by bacteria of the genus *Brucella*, posing a serious threat to global public health and inflicting enormous economic losses in the livestock industry (1). The global annual incidence is conservatively estimated at 2.1 million cases (2), although significant under-reporting is likely in many endemic regions. *Brucella* infects multiple domestic animals and humans and can cause severe outcomes, including prolonged fever, meningitis, spondylitis, arthritis, osteomyelitis, orchitis, endocarditis, liver abscesses, peritonitis, and immune thrombocytopenic purpura (3).

A hallmark of brucellosis is its chronic and relapse-prone nature (4), which presents major challenges for clinical treatment and public health control. The main challenge is Brucella’s ability to establish persistent intracellular infections, as conventional antibiotic therapies often fail to completely eradicate the pathogen residing within host cells (5).

The successful establishment of chronic infection stems from the evolution by *Brucella* of a complex and sophisticated multi-dimensional immune evasion strategy (6). Unlike many pathogens, *Brucella*’s immune intervention is not reliant on a single molecule or pathway but constitutes a dynamically evolving, multi-stage network operating throughout the infection process.

Previous research has identified numerous key virulence factors and their mechanisms. For instance, the Type IV Secretion System (T4SS) secretes effector proteins (e.g., VceA, VceC) to inhibit phagosome-lysosome fusion (7). Its lipopolysaccharide (LPS), featuring a non-classical lipid A structure (e.g., C28 long-chain fatty acids), only weakly activates the TLR4 signaling pathway, thereby minimizing pro-inflammatory cytokine production (8). Furthermore, outer membrane proteins such as Omp25 can inhibit interferon responses by degrading host cGAS, among other methods (9).

Existing reviews often focus on dissecting specific virulence factors or pathways at the molecular level or present the host-pathogen interaction as a relatively static process (6, 10, 11). Isolated discussions of mechanisms at a single infection stage (e.g., intracellular survival) lack systematic organization of the dynamic evolution and spatiotemporal heterogeneity of immune evasion strategies across the entire infection continuum. This limitation hinders a comprehensive understanding of how *Brucella* dynamically “adjusts on demand” its strategies to achieve chronicity.

Therefore, elucidating the spatiotemporal patterns of *Brucella* immune evasion strategies—over time (infection stages) and space (different tissue microenvironments)—is crucial for deciphering the mechanism of chronicity. Accordingly, this review innovatively proposes a four-stage infection model (colonization, latency, acute, chronic) and constructs a “Spatiotemporal Dynamic Immune Evasion Model”. This model systematically elaborates the key immune evasion mechanisms employed by *Brucella* at different stages and their sequential transitions, providing a novel theoretical framework for understanding brucellosis chronicity and laying the groundwork for developing precise, stage-adapted interventions and next-generation vaccines.

Position of this model among previous Brucella immune-evasion reviews: Foundational reviews have been indispensable for cataloguing virulence factors but often lack integration into a staged, dynamic infection process. Seminal works like Atluri et al. (20) provided comprehensive molecular inventories of host-pathogen interactions without organizing them temporally. Later reviews, such as Jiao et al. (19), excelled in mechanism-centric dissection of intracellular parasitism (e.g., autophagy, apoptosis, inflammation) but were structured by biological process rather than temporal stage. Contemporary reviews have begun emphasizing chronicity and immunosuppression; for instance, Pellegrini et al. (12) detailed immunosuppressive mechanisms in chronic brucellosis, and Avila-Calderón et al. (26) focused on interactions with dendritic cells. However, a systematic framework dynamically connecting the initial ‘stealth’ phase to the final ‘manipulation’ phase across a defined spatiotemporal continuum remains lacking. Our four-stage “Spatiotemporal Dynamic Immune Evasion Model” addresses this gap by synthesizing previously isolated insights into a unified narrative, explicitly charting the sequential and strategic shift in immune evasion tactics from colonization to chronicity, thereby offering a novel perspective for understanding and targeting the entire infection course.

## 2 Characteristics and diagnostic criteria for each stage of brucellosis

The chronicity of brucellosis is intimately linked to its unique intracellular parasitism and immune evasion mechanisms. It is crucial to emphasize that the clinical progression of human brucellosis is often fluid and heterogeneous, characterized by delayed onset, diagnosis, and overlapping serological findings. Chronic disease may manifest without clear, sequential transitions through distinct stages. Therefore, the four-stage model proposed herein (**Table 1**) should be interpreted as a conceptual framework based on experimental infections (e.g., murine models) and observed clinical tendencies, rather than a rigid, clinically validated diagnostic system. This model organizes our understanding of the spatiotemporal dynamics of immune evasion, acknowledging that human infections do not always conform neatly to these proposed boundaries. .

**Table 1:** Characteristics of the four stages of Brucella infection.

Stage	Time	Pathogen characteristics	Host characteristics
Colonization	Within 72h post-infection	Mucosal adhesion, internalization via lipid rafts	No significant immune response
Latency	3–14 days	rBCV formation, intracellular replication	No typical clinical symptoms, mild local lymph node enlargement
Acute Phase	2–12 weeks	aBCV-mediated spread, metabolic hijacking	Undulant fever, night sweats, hepatosplenomegaly
Chronic Phase	> 12 weeks	Multi-pathway suppression of host immune response, persistent proliferation and spread	Recurrent symptoms, ineffective antibiotic treatment or relapse

## 3 Immune evasion mechanisms at each stage

The following sections detail the specific immune evasion mechanisms employed during each stage.

### 3.1 Colonization stage

*Brucella* primarily invades the host through the oropharyngeal and genital mucosa and is rapidly internalized by phagocytes (e.g., macrophages, neutrophils, dendritic cells). These cells migrate to secondary and tertiary lymphoid organs, facilitating bacterial spread to nearly all body organs (12).

#### 3.1.1 Adhesion

##### 3.1.1.1 The adhesion process

**Figure 1:**
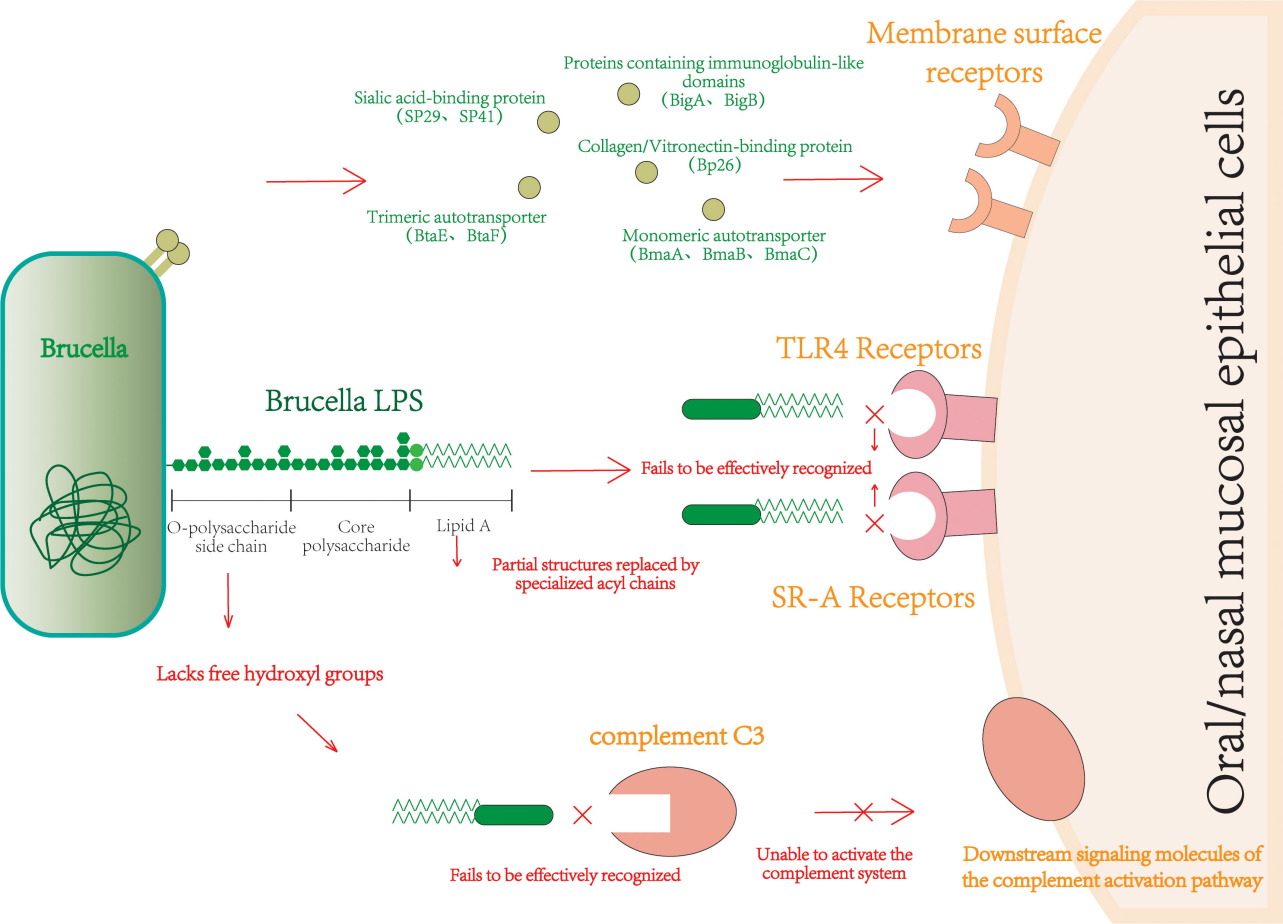
Schematic diagram of *Brucella* ‘locking onto’ the host via adhesins.

*Brucella* adheres to host oral/nasal mucosa via bodily fluids, expressing various adhesin molecules for firm attachment (**Figure 1**). These adhesins include sialic acid-binding proteins SP29 and SP41; immunoglobulin-like domain proteins BigA and BigB; monomeric autotransporters BmaA, BmaB, and BmaC; trimeric autotransporters BtaE and BtaF; and the collagen/vitronectin-binding protein Bp26.

Upon binding to the cell surface, *Brucella* interacts with multiple host receptors. Fc-γ receptor IIa, complement receptor 3 (CR3), and pattern recognition receptors recognize the O-chain fragment of *Brucella* LPS. The class A scavenger receptor (SR-A) recognizes the lipid A structure in LPS. Toll-like receptors (TLR) TLR2, TLR4, and TLR6 detect *Brucella* LPS and lipoproteins, while TLR3, TLR7, and TLR9 recognize nucleic acid motifs (13).

Components of the T4SS, such as VirB5, can function as adhesins located on the bacterial surface, is essential for macrophage infection and acts as a specific adhesin targeting host cell receptors.

Although traditionally considered non-motile, the *Brucella* genome encodes a complete flagellar system. FigJ is a flagellar-associated peptidoglycan hydrolase (located in ORF BABL_0260 of genomic island GI-3). Studies show that a FigJ deletion mutant (*B. abortus* 2308 Δ*figJ*) exhibits significantly reduced biofilm formation capacity (adhesive biomass) on polystyrene plates, indicating FigJ’s direct or indirect role in bacterial adhesion to inert surfaces. Furthermore, this mutant’s survival within macrophages (J774.A1) and epithelial cells (HeLa) significantly decreased, and it failed to effectively colonize the endoplasmic reticulum replicative compartment (rBCV), leading to reduced persistence in mouse spleens. These results collectively indicate that FigJ plays a key role in the colonization stage by promoting adhesion/biofilm formation and subsequent intracellular survival, representing an important novel virulence factor (14).

These adhesin molecules regulate *Brucella* attachment to host cell surface molecules and extracellular matrix components. Besides ensuring firm attachment, they are also viewed as immunogens activating host immunity or potential vaccine candidates (15), as they constitute the first immunogens encountered by the host. An effective adhesin-based vaccine could potentially provide strong and early protection. For example, Deng et al. (16) identified a single-domain antibody, BaV5VH4, that binds *Brucella* VirB5 protein, competitively blocking its interaction with host cell surface receptors and protecting the host.

##### 3.1.1.2 Immune evasion during adhesion

*Brucella* LPS is a key component of its immune evasion arsenal, primarily composed of core polysaccharide, O-polysaccharide side chain, and lipid A. The lipid A is a diaminoglucose disaccharide substituted with C16, C18, C28, and other very long acyl chains. This unique structure prevents effective recognition by TLR4 (17); although it can signal through TLR4, it is only active at very high concentrations (18). Compared to classic enterobacterial LPS (e.g., *E. coli*), *Brucella* LPS induces TNF-α much less efficiently (producing less than 1/10 at the same lipid A concentration) and requires high concentrations (50 nM) to weakly induce p47-GTPases (like IGTP/IIGP). This weak activation is directly linked to its non-classical lipid A structure, confirming its role in attenuating pro-inflammatory responses via the TLR4 pathway through structural modification (18). TLR4 is widely considered the primary receptor complex for LPS binding and signaling. This low-immunogenicity structure means *Brucella* LPS barely induces inflammatory responses in macrophages and DCs (19). *Brucella* lipid A also contains long fatty acid chains (C28), greatly reducing its endotoxin properties (20) and making it less detectable by the host.

The O-chain of bacterial LPS typically has free hydroxyl residues that facilitate complement C3 binding; *Brucella*’s O-chain lacks these free hydroxyls. When complement C3 contacts the specific O-chain of *Brucella* LPS, it cannot be properly cleaved to produce C3a and C3b, thus evading the host’s classical and alternative complement activation pathways (19). This also inhibits neutrophil degranulation, preventing the release of lysosomal substances like myeloperoxidase (MPO), and helping the bacteria avoid immune capture (16, 19). Besides LPS, *Brucella* flagellin also evades TLR5 recognition due to the lack of a specific domain (21).

#### 3.1.2 Cell entry

##### 3.1.2.1 The entry process

Lipid rafts, enriched in glycosphingolipids and cholesterol, facilitate membrane-related processes such as multi-molecular complex formation, transmembrane signaling, and membrane fusion (22). LPS is a key molecule in the interaction between *Brucella* and host cell lipid rafts. It interacts with plasma membrane lipid rafts, promoting *Brucella*-host cell contact and mediating its internalization into phagocytes. It also helps prevent complement-mediated bacterial lysis and host cell apoptosis (23). SR-A and prion protein (PrPc) have been shown to participate in *Brucella* cell invasion via lipid rafts (24). PrPc and SR-A, serving as receptor proteins for heat shock protein 60 (HSP60) and LPS, reside in specific lipid rafts. Disrupting lipid rafts effectively reduces early *Brucella* survival in macrophages, indicating the necessity of lipid raft involvement for early bacterial survival (25).

Additionally, the SP41 protein encoded by the *BMEI0216* gene in *B. melitensis*, the *efp* gene, and virulence island proteins Bab1_2009–2012 can also promote *Brucella* entry into host cells (26).

##### 3.1.2.2 Immune evasion during entry

*Brucella* invades host cells via a lipid raft-dependent endocytic pathway, regulated by LPS and the two-component system BvrR/BvrS (27). Specifically, the O-chain structure of LPS is crucial for raft-mediated internalization. The O-chain and related polysaccharides consist of non-reducing N-formyl-perosamine sugars. These features help reduce the negative charge on the bacterial surface. This special cell envelope structure prevents *Brucella* from binding complement, bactericidal defensins, bacitracin, or other cationic bactericidal molecules, and also protects it against most bactericidal factors like lysosomal extracts, lysozyme, phospholipases, and lactoferrin. The BvrR/BvrS two-component system directly activates the host small GTPase Cdc42 by regulating outer membrane protein (e.g., OMP25/OMP22) expression and lipid A modification, mediating cytoskeleton rearrangement and internalization.

### 3.2 Latency stage

**Figure 2:**
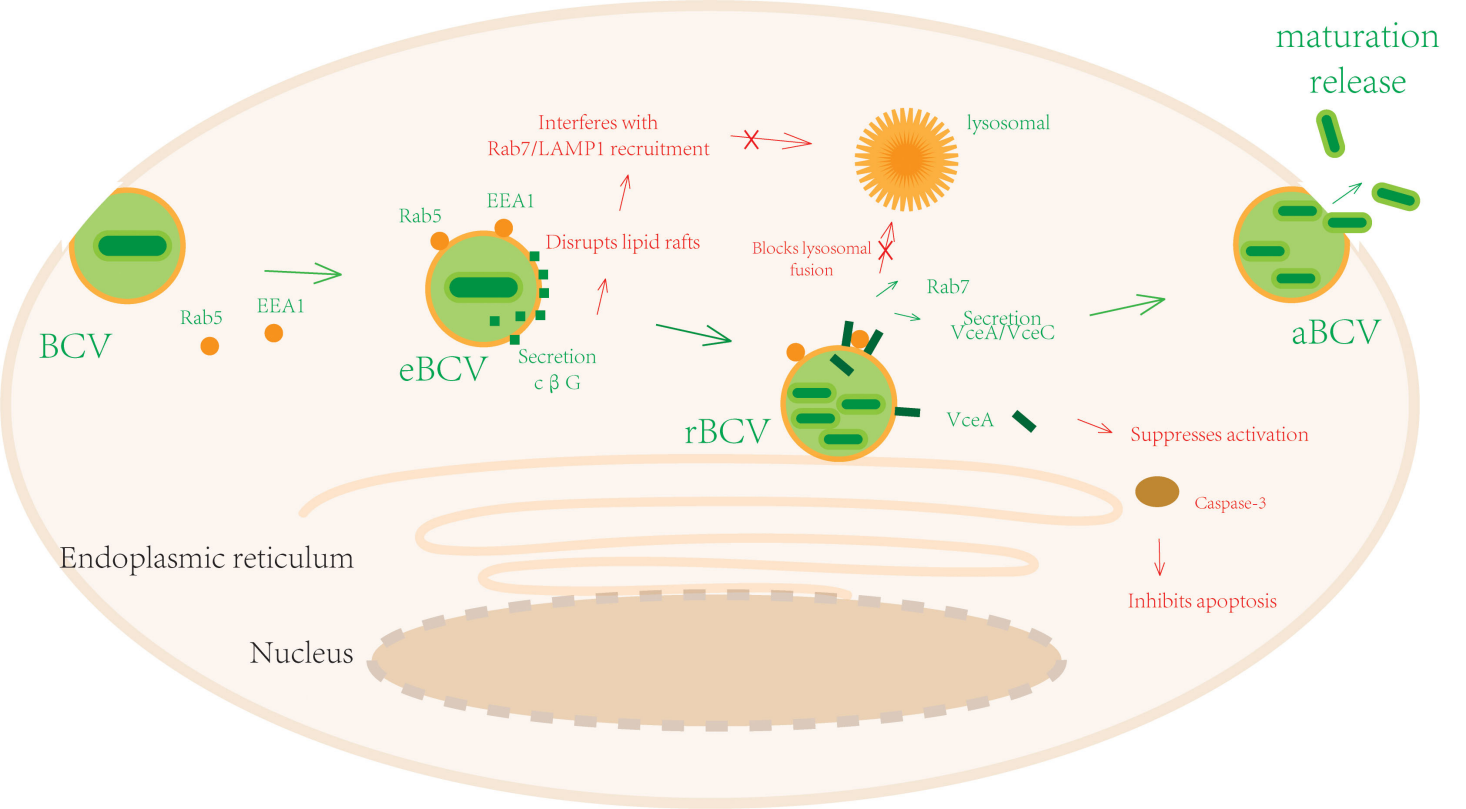
Schematic diagram of *Brucella* immune evasion mechanisms during the latency stage.

After entering the host cell, *Brucella* is initially enclosed within an early *Brucella*-containing vacuole (eBCV). This vacuole is subsequently remodeled into an endoplasmic reticulum-like replicative compartment (rBCV). Upon reaching a critical bacterial density, the rBCV transforms into an autophagic-like vacuole (aBCV). Some bacteria utilize this structure to escape into the cytoplasm, exploit host actin polymerization to form membrane protrusions, and complete the intracellular lifecycle via cell-to-cell spread or host cell lysis to release progeny (**Figure 2**).

#### 3.2.1 Intracellular survival

##### 3.2.1.1 The survival process

After entering the host cell, *Brucella* resides within an early *Brucella*-containing vacuole (eBCV). At this stage, the eBCV briefly associates with early endosome markers (like Rab5, EEA1), initiating phagosome maturation. Subsequently, *Brucella* utilizes the T4SS to secrete VirB effector proteins (like VceA, VceC) to block lysosome acidification enzyme activity and inhibit Rab7-mediated lysosome fusion. Simultaneously, it recruits endoplasmic reticulum (ER)-associated proteins to remodel the eBCV into an ER-like replicative compartment (rBCV). This creates a low-oxygen microenvironment rich in lipids and nutrients, supporting extensive bacterial binary fission using host ER-derived metabolites. Once bacterial numbers saturate, the rBCV transforms into an autophagic-like vacuole (aBCV) by activating autophagy-related pathways or recruiting late endosome markers. Some bacteria escape into the cytoplasm, use host actin polymerization to form membrane protrusions, and complete the intracellular lifecycle via cell-to-cell spread or host cell lysis to release progeny.

##### 3.2.1.2 Immune evasion during survival

To ensure intracellular survival, *Brucella* employs a multifaceted strategy targeting key host cellular processes. A central component is the secretion of cyclic β-1,2-glucan (cβG), which disrupts cholesterol-rich lipid rafts on the *Brucella*-containing vacuole (BCV) membrane. This disruption interferes with BCV maturation and prevents lysosome fusion (28), thereby enabling the remodeling of the early BCV into a replicative compartment (rBCV). The cooperative role of cβG is further supported by observations that even killed *Brucella* can release cβG into the phagosome lumen, aiding the survival of neighboring bacteria (29), and that exogenous cβG can rescue the intracellular survival defect of cβG-deficient mutants (29).

Beyond cβG, the efficient assembly and function of the Type IV Secretion System (T4SS) is critical for intracellular evasion. Recent research demonstrates that UTP–glucose–1–phosphate uridylyltransferase (UCPase), a key metabolic enzyme, positively regulates the transcription of T4SS structural and effector proteins by promoting the expression of ribosomal protein S12 (rpsL), BMEI1825, and 2,4,5-trihydroxyphenylalanine quinone (topA) (30). UCPase deficiency impairs T4SS assembly, reduces effector delivery efficiency, and consequently disrupts rBCV remodeling (30).

The T4SS effector proteins themselves are pivotal instruments of immune evasion. The VirB system blocks the co-localization of the BCV with lysosome markers (such as cathepsin D), preventing lysosome fusion (32, 33) and facilitating BCV conversion into an rBCV. Furthermore, the effector protein VceA plays a distinct role; studies indicate that a *ΔVceA* mutant induces autophagy and inhibits apoptosis in human trophoblast cells, suggesting that the intact VceA protein is crucial for suppressing these host defense mechanisms to enable persistent bacterial survival (31).

This evasion strategy is further reinforced by *Brucella* outer membrane proteins. Omp31 can inhibit TNF-α-induced activation of Caspase-8 and Caspase-9, while Omp25 reduces Caspase-3 activity, collectively blocking apoptosis signals. Simultaneously, these proteins downregulate the expression of the key autophagy protein Beclin 1, which inhibits autophagosome formation and prolongs the intracellular latency of the pathogen (35). Through this coordinated manipulation of apoptosis and autophagy, *Brucella* effectively creates a protected niche for its prolonged survival within the host cell.

#### 3.2.2 Replication

##### 3.2.2.1 The replication process

Inside the rBCV, *Brucella* initiates binary fission proliferation, utilizing host ER-derived erythritol and glucose as carbon sources. When bacterial density reaches a threshold, the rBCV transforms into an aBCV by recruiting autophagy-related proteins like the ATG5-ATG12 complex. The aBCV forms membrane protrusions via actin polymerization, mediating bacterial escape into the cytoplasm or neighboring cells, completing the transition from latency to acute phase.

##### 3.2.2.2 Immune evasion during replication

*Brucella* requires aBCVs to complete its intracellular lifecycle and cell-to-cell spread (36). The host protein Yip1A plays an important role in forming rBCVs and aBCVs. In Yip1A-knockout host cells, *Brucella* cannot form rBCVs and remains trapped in lysosomes. aBCV formation depends on the small GTPase Rab9 (37). When ER Beclin 1 and PI3K form a complex, the rBCV begins converting to aBCV, but aBCV formation gradually decreases as ATG14L is depleted (19). The effector protein BspB interacts with the conserved oligomeric Golgi (COG) complex, regulating COG-dependent trafficking to redirect Golgi-derived vesicles to the BCV, promoting rBCV formation and *Brucella* intracellular proliferation (19).

Essential genes (*manB*, *wboA*) synthesize the LPS O-side chain, necessary for *Brucella* to establish intracellular replication niches (38). Smooth *Brucella* interact with TNF-α via the O-chain, inhibiting host cell apoptosis and thereby promoting their own survival and replication within host cells. Because infected cells do not release apoptosis-specific factors, the immune system is not fully activated, allowing *Brucella* to evade immune surveillance. Notably, besides the T4SS, lytic transglycosylase genes (e.g., BAB_RS22915) play a key role in lysosome escape: a BAB_RS22915 deletion mutant (Δ22915), while retaining LPS O-chain integrity, cannot effectively block fusion of lysosome marker LAMP-1 with bacteria-containing vacuoles (BCVs) in macrophages. This leads to a more than 10-fold drop in intracellular survival rate at 24 hours, and the mutant shows significantly higher co-localization with lysosomes (75%) later in infection, proving loss of lysosome escape ability (39).

Furthermore, *Brucella* disrupts host homeostasis by inhibiting autophagy, an innate immune mechanism. *Brucella* effectors can interfere with host homeostasis by suppressing autophagy. VceA is one of the earliest discovered T4SS substrates, regulated by VjbR, and highly conserved in all sequenced *Brucella* genomes.

The ubiquitin-proteasome system (UPS) and autophagy-lysosome pathway (ALP) are two major protein degradation pathways in eukaryotic cells. In *Brucella suis*-infected mouse macrophages, increased expression of autophagy marker LC3-II, increased autophagosome formation, and incomplete suppression of P62 expression were observed. Moreover, treatment with the autophagy inhibitor 3-methyladenine, which simultaneously inhibits UPS and ALP, severely impaired *Brucella suis* intracellular proliferation (40).

Although T4SS is crucial for intracellular survival in mammalian models, its role differs in the *Galleria mellonella* (wax moth) model (17), where a VirB-T4SS deficient strain (BaΔVirB) did not show significantly impaired intracellular replication, suggesting *Brucella* might use alternative, non-classical host immune regulation pathways for escape.

Emerging research found that the T4SS effector protein BspF can regulate lysine crotonylation (Kcr) modifications on host proteins, suggesting a potential role in promoting intracellular replication (41). BspF itself has decrotonylase activity, and its overexpression causes widespread changes to the host crotonylome. Studies suggest that crotonylation modifications on specific host proteins like Rab9A and RAP1B might affect *Brucella* intracellular survival, potentially by remodeling the BCV microenvironment and inhibiting host immune recognition. While this represents an exciting and novel mechanism of immune modulation, its functional significance across the entire infection spectrum is still an emerging area of investigation.

### 3.3 Acute phase

**Figure 3:**
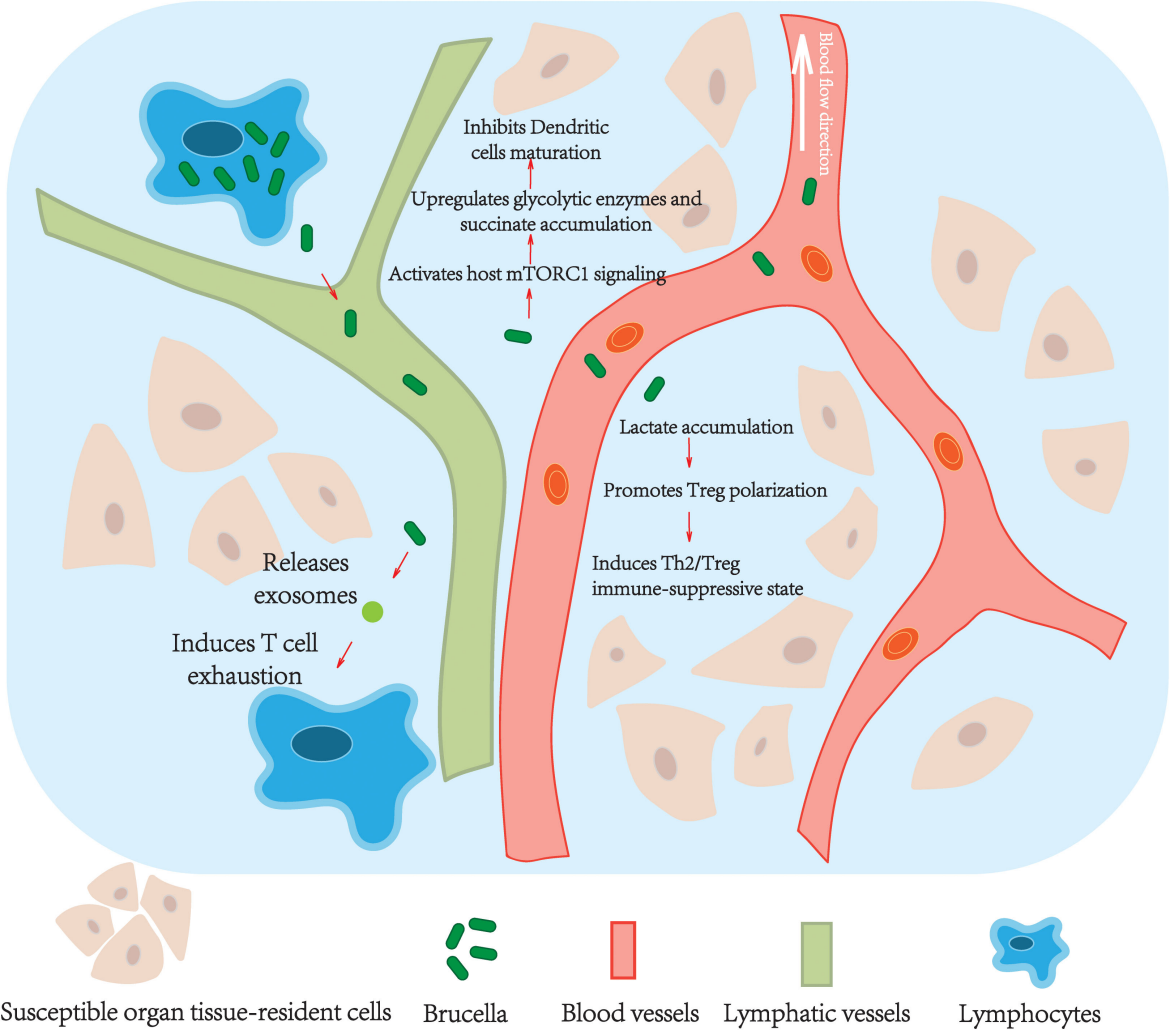
Schematic diagram of *Brucella* immune evasion mechanisms during the acute phase.

Upon entering the acute phase, *Brucella*’s immune evasion strategies shift toward systemic dissemination and active suppression of host immune responses (**Figure 3**). This stage is characterized by the pathogen breaking out of local infection sites, using host cells as cellular carriers, and establishing a dissemination-suppression dual regulatory network through metabolic hijacking—the manipulation of host cell metabolic pathways (e.g., accumulation of succinate) to suppress immune function—and immune signal interference.

#### 3.3.1 Cell-to-cell migration

##### 3.3.1.1 The migration process

To facilitate understanding of the intracellular progression leading to aBCV formation and cell-to-cell spread, the key transitions are summarized below. This process initiates during the latency stage and culminates in dissemination during the acute phase (**Table 2**).

**Table 2:** Key transitions in the Brucella intracellular lifecycle leading to cell-to-cell spread.

Stage	Vacuole form & process	Key function & evasion mechanism
Early Latency	eBCV (early BCV)	Avoiding lysosomal killing
	Phagosome maturation initiates	T4SS effectors block lysosome fusion
Mid-Latency	rBCV (replicative BCV)	Creating a replicative niche
	ER-derived niche	Sequestration from immune surveillance nutrient acquisition
	Massive bacterial replication	
Late Latency / Acute Phase	aBCV (autophagic-like BCV)	Mediating cell-to-cell spread
	Recruits autophagy-related proteins	Enables systemic dissemination while remaining intracellular
	Forms actin-based protrusions	

Following the transformation of rBCVs into autophagic-like vacuoles (aBCVs) in the late latency stage, as described in section 3.2.2.1, these aBCVs form membrane protrusions via actin polymerization. This process mediates direct *Brucella* penetration into adjacent cells. Some bacteria are released extracellularly through host cell lysis, disguised as “self” components within phosphatidylserine (PS)-labeled apoptotic bodies, and are phagocytosed by surrounding macrophages, thus achieving cell-to-cell spread.

##### 3.3.1.2 Immune evasion during migration

*Brucella* employs a multi-dimensional strategy to control neutrophil function and death mode, forming a “Trojan horse” immune evasion and communication strategy (42). After internalization into neutrophils via TLR- and complement receptor-mediated pathways, LPS inhibits the respiratory burst by bacterial secretion of superoxide dismutase and catalase, and interferes with calcium signaling and SNARE complex-dependent membrane fusion, thus blocking degranulation and lysosomal bactericidal functions. Simultaneously, *Brucella* specifically inhibits NADPH oxidase activity, hindering neutrophil extracellular trap (NET) formation, and induces caspase-dependent apoptosis instead of NETosis. This causes premature neutrophil death with surface exposure of PS, which is recognized and phagocytosed by macrophages via MerTK/Tim-4 receptors. During this process, *Brucella* internalized within neutrophils is delivered into macrophages where it survives, turning these macrophages into “Trojan horses” that disseminate via the lymphatic system to other host tissues.

#### 3.3.2 Tissue-to-tissue dissemination

##### 3.3.2.1 The dissemination process

*Brucella* migrates to target organs like the liver, spleen, and joints via lymphatic vessels or blood circulation. Monocytes and dendritic cells (DCs) act as primary carriers, transporting the pathogen across vascular endothelial barriers and using chemokine receptors (e.g., CCR7) for directed migration to secondary lymphoid tissues, establishing secondary infection sites. Research has identified dissemination from cervical lymph nodes (CNL) to other tissues (26). Additionally, *Brucella* uses platelet adhesion and endothelial cell invasion to cross the blood-brain barrier or placental barrier, ultimately colonizing immune-privileged organs (e.g., meninges, testes) (26).

##### 3.3.2.2 Immune evasion during dissemination

*Brucella* exploits B cell receptor (BCR) specificity to enhance host susceptibility (43). Studies found that when B cells cannot recognize *Brucella* antigens via their BCR (e.g., in MD4 transgenic mice expressing a BCR for an unrelated antigen, HEL), host resistance to *Brucella* infection significantly increased, and B cell uptake efficiency of *Brucella* significantly decreased. This indicates that specific recognition of *Brucella* antigens by the BCR promotes bacterial entry into B cells, ultimately making the host more susceptible. Importantly, the inability to secrete antibodies (in sIgM^-^/AID^-^ mice) did not alter host resistance or *Brucella* load in B cells, showing this BCR-mediated enhanced susceptibility is independent of antibody secretion.

*Brucella* outer membrane protein Omp25 promotes ubiquitination of the host intracellular cyclic GMP-AMP synthase (cGAS), leading to its degradation via the proteasome pathway (44). This degradation reduces cGAS enzymatic activity when cells are stimulated by DNA viruses (e.g., pseudorabies virus PRV, herpes simplex virus type 1 HSV-1, porcine parvovirus PPV, bovine herpesvirus type 1 BoHV-1) or interferon-stimulatory DNA (ISD), significantly decreasing production of its catalytic product, cyclic GMP-AMP (cGAMP).

Due to cGAS degradation by Omp25 and reduced activity, phosphorylation levels of the key downstream adaptor protein STING (stimulator of interferon genes) significantly decrease. Phosphorylation of interferon regulatory factor 3 (IRF3) is also inhibited, and nuclear translocation of phosphorylated IRF3 (p-IRF3) is reduced. This blocks effective activation of the cGAS-STING-IRF3 signaling pathway in experimental models.

Collectively, these *in vitro* findings (44) suggest that Omp25-mediated inhibition of the cGAS-STING-IRF3 pathway may contribute to immune evasion by suppressing Type I interferon production (particularly interferon beta, IFN-β), thereby potentially facilitating early systemic dissemination. Its role as a central driver of chronic infection, however, requires further validation *in vivo*.

The final consequence of Omp25 inhibiting the cGAS-STING-IRF3 pathway is significant suppression of Type I interferon production (particularly IFN-β) after host cells detect cytosolic DNA (from viruses or other stimuli). Transcriptional expression levels of interferon-stimulated genes (ISGs) induced downstream of IFN-β, such as ISG56 and the chemokine IP-10 (CXCL10), are also markedly reduced. This weakens the host cell’s antiviral innate immune response.

VceC can induce endoplasmic reticulum (ER) stress and the unfolded protein response (UPR) (34), promoting IL-6 and TNF-α release, leading to granuloma formation, which favors persistent bacterial infection.

### 3.4 Chronic phase

When *Brucella* infection enters the chronic phase, its core strategy is to systemically establish and maintain an immunosuppressive microenvironment conducive to its long-term survival. This involves the pathogen actively implementing multiple immunosuppressive strategies, ultimately leading to severe dysfunction of the host immune response.

#### 3.4.1 Suppression of innate immunity

*Brucella* can interfere with specific T cell recognition through molecular mimicry: Its proteins contain “neighbor” pentapeptide motifs (e.g., KSINAERL, PQKINIDRT) structurally similar to the model antigen SIINFEKL. These can cross-activate transgenic OT-1 TCR T cells (whose TCR should specifically recognize SIINFEKL-H2K^b^ complexes). Although this cross-reaction has low affinity and is peptide concentration-dependent, it is sufficient to induce IFN-γ secretion and cytotoxic responses during infection. Genome-wide analysis revealed *Brucella* carries 38 such motifs, widely distributed in outer membrane proteins (OMPs) and various metabolic enzymes. These may weaken immune surveillance by mimicking host peptides and provide a molecular basis for autoimmune phenomena common in chronic infection (45).

*Brucella abortus* RNA can reduce IFN-γ-induced MHC-I molecule expression on the surface of human monocytes (46). This is not due to reduced MHC-I synthesis but rather retention of MHC-I molecules within the Golgi apparatus. The authors also confirmed that the MHC-I proteins retained in the Golgi are correctly assembled. *Brucella* achieves early MHC-I inhibition by activating the EGFR-ERK signaling axis, a mechanism independent of classic virulence factors. Studies found MHC-I surface downregulation detectable as early as 8 hours post-infection (peaking at 48 hours), and mutants lacking VirB T4SS or LPS O-chain (RB51, ΔvirB10) retained this ability. The pathogen induces host secretion of ligands like EGF and TGF-α, which activate EGFR and ErbB2 via autocrine/paracrine signaling, triggering an ERK1/2 phosphorylation cascade, ultimately causing MHC-I retention in the Golgi. Key evidence includes: using EGFR blocking antibody (cetuximab), ErbB2 inhibitor (trastuzumab), or TACE protease inhibitor (GM6001) partially reversed MHC-I suppression; exogenous EGF/TGF-α could replicate the effect; infected supernatant could also suppress MHC-I expression in uninfected cells (47).

Evidence from clinical cohorts, while often limited in sample size, provides initial insights into inflammasome activity in human brucellosis. For instance, a study profiling peripheral blood mononuclear cells (PBMCs) from a defined cohort of acute and chronic brucellosis patients (48) reported that AIM2 inflammasome expression was significantly elevated in acute cases, while it was significantly lower in chronic patients. Based on these observations from a specific patient population, it has been proposed that Brucella may, in some contexts, weaken DNA recognition efficiency by suppressing AIM2 signaling while hijacking Caspase-1 activity, potentially creating a ‘low-responsive inflammatory steady state’ conducive to persistence. However, given the heterogeneity of chronic brucellosis, the universality of this mechanism across all patients requires further validation in larger, diverse cohorts.

*Brucella* infection activates the host PI3K/AMPK/Nrf2 signaling pathway, leading to the sustained upregulation of heme oxygenase-1 (HO-1). Notably, the immunosuppressive effect of HO-1 is mediated not by its enzymatic metabolites (e.g., biliverdin, CO) but through the direct suppression of key macrophage bactericidal functions, including a significant reduction in the production of reactive oxygen species (ROS), TNF-α, and IL-1β. The critical role of this pathway is demonstrated by the fact that HO-1 gene knockout (HO-1^-^/^-^) or pharmacological inhibition with tin protoporphyrin (SnPP) significantly reduces intracellular bacterial load, whereas HO-1 induction with cobalt protoporphyrin (CoPP) exacerbates infection in both macrophages and mouse models (41, 49). Thus, HO-1 represents a key host factor exploited by *Brucella* to establish chronic infection by blunting the innate immune response.

Additionally, *Brucella* secretes heat-stable small molecules (<1000 Da, containing nucleotide-like substances) that specifically inhibit the myeloperoxidase (MPO)-mediated iodination reaction, blocking the MPO-H_2_O_2_-halide system’s bactericidal function (50). This inhibition occurs by preventing degranulation, not by directly interfering with H_2_O_2_ production or MPO enzyme activity.

*Brucella* suppresses IFNγ signaling in the chronic phase by disrupting the STAT1–CBP/P300 transcription complex. Infection does not block STAT1 tyrosine phosphorylation (Tyr701) or nuclear translocation but activates the host cAMP/PKA pathway, inducing sustained CREB phosphorylation at Ser133. Phosphorylated CREB competitively binds the transcriptional coactivators CBP/P300, preventing IFNγ-activated STAT1 from effectively recruiting them. This specific complex dissociation selectively inhibits CBP/P300-dependent IFNγ response genes (e.g., FcγR1/CD64), thereby weakening macrophage bactericidal function and antigen presentation efficiency, without affecting baseline STAT1 activation. This creates an immune-tolerant environment for chronic infection (51).

Furthermore, *Brucella* utilizes the ABC transporter system YejABEF to resist host antimicrobial peptide (AMP) killing, enhancing survival within macrophages. Studies show YejABEF (especially the membrane protein YejE) is induced by polymyxin B; its deletion mutants (ΔyejE and ΔyejABEF) show significantly increased sensitivity to acidic stress and AMPs, leading to impaired replication in macrophages and a spleen bacterial load drop of over 2 log in mouse infection models. This transporter system provides a key defense barrier for *Brucella* long-term survival under immune pressure by maintaining cell membrane integrity against the bactericidal action of AMPs within lysosomes (52).

#### 3.4.2 Suppression of adaptive immunity

*Brucella* targets dendritic cell (DC) function through specific outer membrane proteins (e.g., Omp25 and Omp31) to establish an immunosuppressive microenvironment (53). Omp25 and Omp31 significantly inhibit maturation of mouse bone marrow-derived dendritic cells (BMDCs), downregulate co-stimulatory molecules CD40, MHC-I, and MHC-II expression, induce anti-inflammatory cytokines IL-10 and IL-4 secretion, and simultaneously inhibit pro-inflammatory cytokines TNF-α, IFN-γ, and IL-12 production. This impairs BMDC antigen presentation function and inhibits T cell proliferation, creating a Th2-type response bias. Further research found Omp25 directly downregulates MHC-II expression by interfering with STAT1 phosphorylation, while Omp31 likely inhibits NF-κB activation through an unknown signaling pathway, jointly blocking host adaptive immune initiation. These mechanisms align with *Brucella* chronic phase immune evasion strategies, weakening host clearance capacity by inhibiting DC-mediated T cell activation, providing an immune-tolerant microenvironment for long-term pathogen latency. *Brucella* inhibits TNF-α secretion in DCs via Omp25, blocking their maturation and antigen presentation function. Human DCs infected with *Brucella* wild-type (WT) show significantly inhibited TNF-α secretion (concentration only 1/10th of the *E. coli* infected group), leading to lack of expression of DC maturation markers (CD83, CCR7, CD40, CD86, HLA-ABC/D) and inability to activate naive CD4^+^ T cell proliferation. Adding exogenous TNF-α restores the maturation phenotype and antigen presentation capacity of infected DCs. Infection with OMP25-deficient or bvrR mutant strains (lacking OMP25 expression) leads to significantly higher TNF-α secretion in DCs, inducing DC maturation and T cell activation; this effect can be completely blocked by anti-TNF-α antibody. Additionally, OMP25-deficient strain-infected DCs secrete more IL-12p70, suggesting they may promote a Th1 immune response (54).

*Brucella abortus* causes CD4^+^ and CD8^+^ T cell immunosuppression by recruiting PD-L1^+^ Sca-1^+^ neutrophils that secrete IL-1RA (42). This immunosuppression does not depend on IL-1 but relies on persistent stimulation by the core polysaccharide of *Brucella* LPS. Furthermore, most PD-L1^+^ Sca-1^+^ myeloid cells are also positive for LAG-3, a well-known T cell inhibitory receptor. The team also found that expression of CD274/PDL1, LAG3, and Ly6E (the putative human homolog of Sca-1) was upregulated in the whole blood of acute brucellosis patients. This finding, derived from human patient samples, supports the potential relevance of the PD-1/PD-L1 axis and LAG-3 in human disease. Correspondingly, and consistent with a state of immune dysregulation, significant upregulation of PD-1 was detected on the surface of CD4^+^ and CD8^+^ T cells in the blood of acute and chronic brucellosis patients. It is important to note that these human data, while highly valuable, represent observations from a specific study population, and the functional impact and heterogeneity of these markers across the broader spectrum of brucellosis patients remain active areas of investigation.

*Brucella* LPS can persist within host cells for months, slowly trafficking intracellularly to enrich in MHC-II compartments, and forming large domains on the cell surface composed of lipid rafts, MHC-II, and Br-LPS. These domains sequester MHC-II–peptide complexes, hindering T cell receptor recognition and thus inhibiting CD4^+^ T cell activation. This mechanism is independent of cytokine regulation and is a key epitope disguise strategy for *Brucella* to establish chronic immune tolerance (29).

Similarly, plant polysaccharides (e.g., nCKAP-2 from *Curcuma kwangsiensis*) can induce MDSC apoptosis and downregulate ROS levels by activating TLR4-NF-κB signaling, thereby reversing MDSC-mediated T cell suppression. This strategy offers a new approach for targeting and clearing immunosuppressive cells accumulated during chronic *Brucella* infection (55).

## 4 Spatio-temporal dynamic immune evasion model

**Figure 4:**
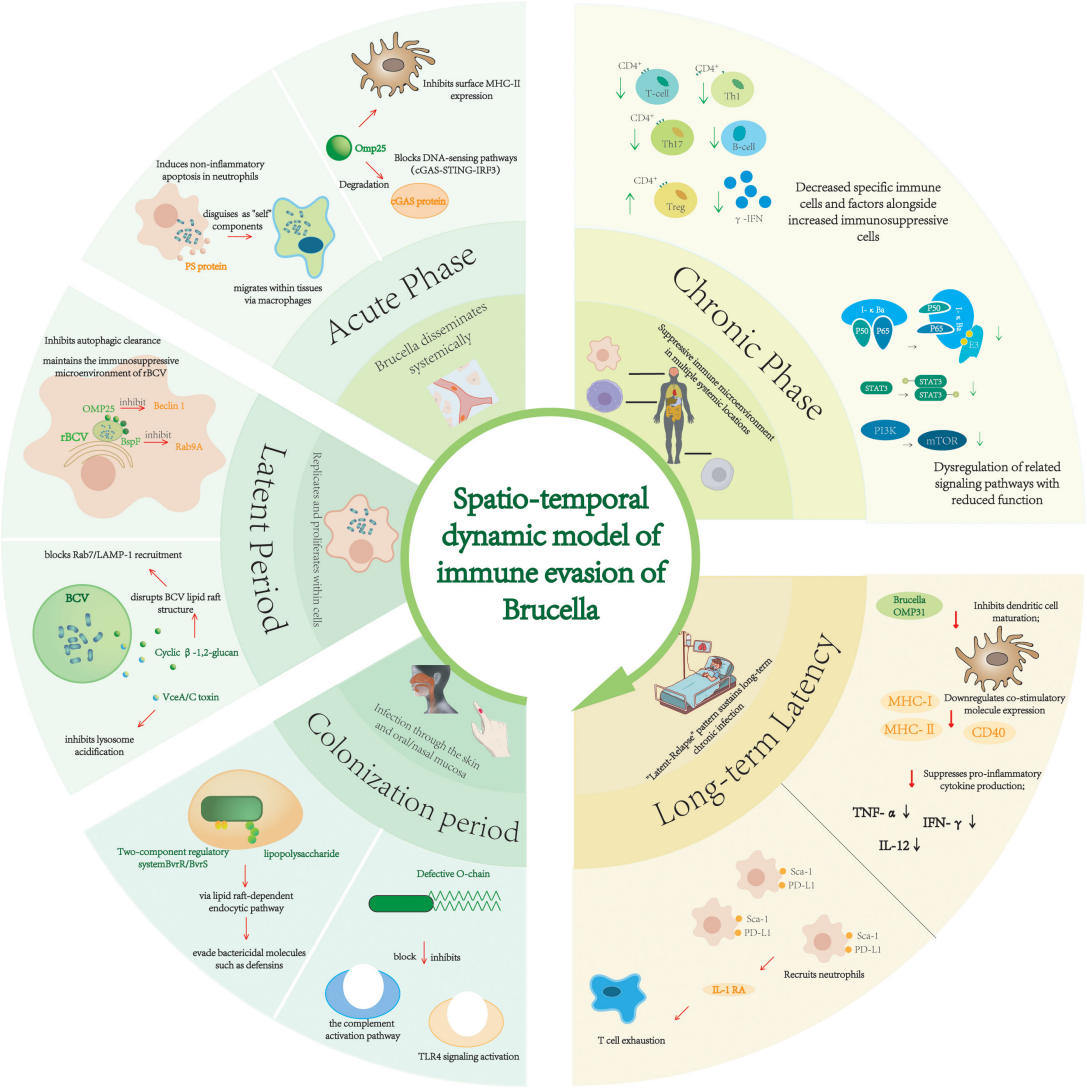
Model of the spatio-temporal dynamics of Brucella immune evasion.

The chronic nature of *Brucella* infection essentially results from its dynamic regulation of the host defense system through multi-stage, multi-dimensional immune evasion strategies. Based on a systematic analysis of the entire infection process, this paper proposes the concept of a “Spatio-Temporal Dynamic Immune Evasion Model.” It divides *Brucella* immune regulation into four stages—Colonization, Latency, Acute, and Chronic—and reveals the core evasion mechanisms at each stage (**Figure 4**).

This model illustrates selected Brucella immune evasion strategies across stages: Adhesion, Cell Entry, Intracellular Survival, Replication, Cell-to-Cell Migration, Tissue Dissemination, Chronic Suppression, and Long-Term Persistence.

In the Colonization stage, the pathogen delays innate immune response initiation by using a low-immunogenicity LPS lipid A structure (C16–C28 acyl chains) to suppress TLR4 signaling activation and an O-chain lacking hydroxyl groups to block complement C3 cleavage. Simultaneously, the T4SS-secreted VirB5 adhesin targets host cell receptors (e.g., Fc-γRIIa), promoting bacterial internalization and hijacking the lipid raft-dependent endocytic pathway for initial colonization. Upon entering Latency, *Brucella* inhibits lysosome acidification enzyme activity and blocks phagosome-lysosome fusion via T4SS effector proteins (e.g., VceA, VceC). It also secretes cyclic β-1,2-glucan (cβG) to disrupt BCV lipid raft structure, remodeling the BCV into an rBCV and creating an immune-privileged microenvironment. Meanwhile, the RicA protein hijacks the host Rab2 GTPase to regulate endomembrane trafficking, ensuring nutrient uptake and escape from autophagy. In the Acute phase, the pathogen achieves transcellular spread via the “Trojan horse” strategy using apoptotic neutrophils and uses outer membrane vesicles (OMVs) to deliver nucleases degrading antimicrobial peptides, suppressing local inflammation. Metabolic hijacking (e.g., succinate accumulation inhibiting DC maturation via SUCNR1) and T cell exhaustion induced by exosomal circRNA-Bruc1 further weaken adaptive immunity. Progressing to the Chronic phase, *Brucella* systemically establishes an immunosuppressive microenvironment through epigenetic reprogramming (e.g., sRNA-16-mediated H3K27me3 modification silencing TNF-α, miR-21 activating PI3K/Akt/mTOR pathway promoting Treg polarization) and OMP25-mediated MHC-II downregulation, achieving long-term latency.

The core value of this model lies in integrating the bidirectional interaction network between pathogen and host, revealing the temporal logic of *Brucella*’s shift from “immune stealth” to “immune manipulation.” The model is strongly supported by single-cell studies which delineate a clear transition from an acute phase dominated by monocyte-driven inflammatory storms (56) to a chronic phase characterized by widespread T and NK cell exhaustion and type I interferon-mediated susceptibility (57). Compared to the traditional “latency-activation alternation” theory, this study is the first to clarify the dynamic transition of stage-specific evasion nodes, such as the functional expansion of the T4SS system from initial adhesion (VirB5) to intracellular survival (VceA/C), and the synergistic effects of metabolic hijacking and epigenetic regulation.

## 5 Therapeutic and prophylactic implications of the spatiotemporal model

The Spatiotemporal Dynamic Immune Evasion Model not only provides a framework for understanding pathogenesis but also unveils a spectrum of opportunities for developing stage-specific interventions. The rationale is that targeting a mechanism critical for a specific stage could disrupt the infection cycle at a vulnerable point, potentially improving efficacy and reducing off-target effects. The following table (**Table 3**) synthesizes potential strategic directions based on this model, linking specific stages to realistic targets, the current level of evidence, and conceivable therapeutic or prophylactic modalities.

**Table 3:** Potential. stage-specific intervention strategies inferred from the Spatiotemporal Dynamic Immune Evasion Model.

Possible modality	Prophylactic vaccine subunit vaccine containing key adhesins	Therapeutic (small molecule) T4SS inhibitor; Host-directed therapy promoting lysosome fusion	Therapeutic (biologic/small molecule) Inhibitor of efferocytosis; STING agonist to restore IFN-I	Therapeutic (immunotherapy) Immune checkpoint blockade (αPD-1/αPD-L1); DC-activating adjuvant
Evidence Level & Key References	Preclinical Monoclonal antibody (BaV5VH4) blocks adhesion *in vitro* (16)	Preclinical / Hypothetical Genetic deletion of effectors impairs intracellular survival (31, 33)	Preclinical Characterized neutrophil apoptosis and uptake (42) Omp25 degrades cGAS *in vitro* (44)	Preclinical / Early Clinical Correlation PD-1^+^ T cells and PD-L1^+^ myeloid cells found in patients (42) Omp25 inhibits DC maturation *in vitro* (53, 54)
Realistic Target / Strategy	Bacterial adhesins (e.g., VirB5)	T4SS effector function (e.g., VceA, VceC) rBCV integrity (e.g., via host pathways)	"Trojan horse" formation cGAS-STING pathway inhibition (Omp25)	T cell exhaustion (PD-1/PD-L1, LAG-3) Immunosuppressive microenvironment (e.g., Omp25-mediated MHC-II suppression)
Infection Stage	Colonization	Latency	Acute Phase	Chronic Phase

It is crucial to acknowledge the challenges in translating these concepts. These include the need for targeted delivery systems to reach specific intracellular niches, the risk of inducing immunopathology by disrupting immune homeostasis, and the inherent heterogeneity of human brucellosis. Nevertheless, by providing a structured roadmap, this spatiotemporal model firmly grounds the promising yet preliminary translational prospects mentioned throughout the review and charts a course for future investigative efforts aimed at achieving precise, stage-adapted control of brucellosis.

## 6 Conclusion

This review, by systematically integrating research findings from the past decade, proposes for the first time a “Spatiotemporal Dynamic Immune Evasion Model” for *Brucella* infection. The core conclusion of this model is that the chronicity of *Brucella* is not the result of a single static mechanism, but rather a comprehensive reflection of its spatiotemporally dynamic switching of immune intervention targets at different stages of infection (colonization, latency, acute, chronic). In the early stage of infection (colonization phase), the pathogen passively “hides” itself relying on its atypical LPS structure, delaying innate immune recognition; upon entering the latency phase, it actively utilizes tools like T4SS effector proteins to remodel intracellular vesicles, creating an “immune-privileged” replication niche; by the acute dissemination phase, the strategy escalates to using tactics like the “Trojan horse” for intercellular spread and systemic dissemination; finally, in the chronic phase, through multi-dimensional mechanisms such as epigenetic reprogramming, suppression of antigen presentation, and induction of T cell exhaustion, it actively “manipulates” the host immune system to establish an immunosuppressive microenvironment conducive to its long-term latency. This is supported by findings that Brucella infection triggers global chromatin reorganization, characterized by reduced long-range contacts and enhanced local interactions, which facilitate the transcriptional activation of immune genes and reflect a key epigenetic strategy for immune evasion (58). This model breaks through the static perspective of the traditional “latency-activation alternation” theory, reveals the synergy and functional expansion of functional modules like the T4SS across different stages, and provides a new, systematic framework for understanding the immunological basis of chronic *Brucella* infection.

Although this model provides an integrative perspective, several limitations remain. First, the stage division of the model is primarily based on cell and animal experiments; its applicability in different human immune cell subsets and tissue microenvironments, as well as the clarity of stage boundaries, still require validation using clinical samples and technologies like spatial transcriptomics and single-cell sequencing. Second, research on the post-translational modifications of host proteins mediated by many key effector proteins (e.g., BspF) is still in its infancy, and their conservation and function across different clinical isolates urgently need support from genetic and biochemical evidence. Finally, many of the temporal nodes and target interactions proposed in the model still lack support from real-time dynamic observation data. Future research should focus on using technologies like live-cell imaging and *in vivo* real-time monitoring to achieve more precise stage division and mechanism validation of the infection process within the body. Looking ahead, exploring stage-specific targeted therapeutic strategies aimed at these key temporal nodes (for example, blocking “Trojan horse” formation in the acute phase, reversing T cell exhaustion or epigenetic silencing in the chronic phase) will lay the foundation for developing precision therapies adapted to the infection process and ultimately provide new ideas for overcoming the challenge of chronic *Brucella* infection.

In summary, this study innovatively proposes the “Spatiotemporal Dynamic Immune Evasion Model”, systematically elaborates the key immune evasion mechanisms employed by *Brucella* at different stages of the infection process and their sequential transition pathways, and provides a theoretical framework for a comprehensive understanding of the immunological basis of chronic *Brucella* infection and the development of novel intervention strategies.

## References

[1] Qureshi KA Parvez A Fahmy NA Abdel Hady BH Kumar S Ganguly A . Brucellosis: epidemiology, pathogenesis, diagnosis and treatment-a comprehensive review. Ann Med. (2024) 55:2295398. doi: 10.1080/07853890.2023.2295398, PMID: 38165919 PMC10769134

[2] Laine CG Johnson VE Scott HM Arenas-Gamboa AM . Global estimate of human brucellosis incidence. Emerg Infect Dis. (2023) 29:1789–97. doi: 10.3201/eid2909.230052, PMID: 37610167 PMC10461652

[3] Tian T Zhu Y Shi J Shang K Yin Z Shi H . The development of a human Brucella mucosal vaccine: What should be considered? Life Sci. (2024) 355:122986. doi: 10.1016/j.lfs.2024.122986, PMID: 39151885

[4] Mode S Ketterer M Québatte M Dehio C . Antibiotic persistence of intracellular Brucella abortus. PloS Negl Trop Dis. (2022) 16:e0010635. doi: 10.1371/journal.pntd.0010635, PMID: 35881641 PMC9355222

[5] Głowacka P Żakowska D Naylor K Niemcewicz M Bielawska-Drózd A . Brucella - virulence factors, pathogenesis and treatment. Pol J Microbiol. (2018) 67:151–61. doi: 10.21307/pjm-2018-029, PMID: 30015453 PMC7256693

[6] Wei Z Zhang S Wang X Bai J Wang H Yang Y . Beyond survival to domination: Brucella’s multilayered strategies for evading host immune responses. Front Microbiol. (2025) 16:1608617. doi: 10.3389/fmicb.2025.1608617, PMID: 40606156 PMC12213604

[7] Yin Y Tian M Zhang G Ding C Yu S . A novel Brucella T4SS effector RS15060 acts on bacterial morphology, lipopolysaccharide core synthesis and host proinflammatory responses, which is beneficial for Brucella melitensis virulence. Microbiol Res. (2024) 292:128015. doi: 10.1016/j.micres.2024.128015, PMID: 39689431

[8] Flores-Concha M Gómez LA Soto-Shara R Molina RE Coloma-Rivero RF Montero DA . Brucella abortus triggers the differential expression of immunomodulatory lncRNAs in infected murine macrophages. Front Immunol. (2024) 15:1352306. doi: 10.3389/fimmu.2024.1352306, PMID: 38464511 PMC10921354

[9] Li R Liu W Yin X Zheng F Wang Z Wu X . Brucella spp. Omp25 Promotes Proteasome-Mediated cGAS Degradation to Attenuate IFN-β Production. Front Microbiol. (2021) 12:702881. doi: 10.3389/fmicb.2021.702881, PMID: 34394047 PMC8358459

[10] Qin Y Zhou G Jiao F Cheng C Meng C Wang L . Brucella mediates autophagy, inflammation, and apoptosis to escape host killing. Front Cell Infect Microbiol. (2024) 14:1408407. doi: 10.3389/fcimb.2024.1408407, PMID: 39507949 PMC11537862

[11] Jiao H Zhou Z Li B Xiao Y Li M Zeng H . The mechanism of facultative intracellular parasitism of brucella. Int J Mol Sci. (2021) 22:3673. doi: 10.3390/ijms22073673, PMID: 33916050 PMC8036852

[12] Pellegrini JM Gorvel J-P Mémet S . Immunosuppressive mechanisms in brucellosis in light of chronic bacterial diseases. Microorganisms. (2022) 10:1260. doi: 10.3390/microorganisms10071260, PMID: 35888979 PMC9324529

[13] Yu H Gu X Wang D Wang Z . Brucella infection and Toll-like receptors. Front Cell Infect Microbiol. (2024) 14:1342684. doi: 10.3389/fcimb.2024.1342684, PMID: 38533384 PMC10963510

[14] Coloma-Rivero RF Gómez L Alvarez F Saitz W Del Canto F Céspedes S . The role of the flagellar protein flgJ in the virulence of brucella abortus. Front Cell Infect Microbiol. (2020) 10:178. doi: 10.3389/fcimb.2020.00178, PMID: 32411617 PMC7198779

[15] Huy TXN Nguyen TT Kim H Reyes AWB Kim S . Brucella phagocytosis mediated by pathogen-host interactions and their intracellular survival. Microorganisms. (2022) 10:2003. doi: 10.3390/microorganisms10102003, PMID: 36296279 PMC9610446

[16] Deng H Zhou J Gong B Xiao M Zhang M Pang Q . Screening and identification of a human domain antibody against Brucella abortus VirB5. Acta Trop. (2019) 197:105026. doi: 10.1016/j.actatropica.2019.05.017, PMID: 31103700

[17] Elizalde-Bielsa A Aragón-Aranda B Loperena-Barber M Salvador-Bescós M Moriyón I Zúñiga-Ripa A . Development and evaluation of the Galleria mellonella (greater wax moth) infection model to study Brucella host-pathogen interaction. Microb Pathog. (2022) 174:105930. doi: 10.1016/j.micpath.2022.105930, PMID: 36496059

[18] Lapaque N Takeuchi O Corrales F Akira S Moriyon I Howard JC . Differential inductions of TNF-alpha and IGTP, IIGP by structurally diverse classic and non-classic lipopolysaccharides. Cell Microbiol. (2006) 8:401–13. doi: 10.1111/j.1462-5822.2005.00629.x, PMID: 16469053

[19] Jiao H Zhou Z Li B Xiao Y Li M Zeng H . The mechanism of facultative intracellular parasitism of brucella. Int J Mol Sci. (2021) 22:3673. doi: 10.3390/ijms22073673, PMID: 33916050 PMC8036852

[20] Atluri VL Xavier MN de Jong MF den Hartigh AB Tsolis RM . Interactions of the human pathogenic Brucella species with their hosts. Annu Rev Microbiol. (2011) 65:523–41. doi: 10.1146/annurev-micro-090110-102905, PMID: 21939378 PMC13363517

[21] Andersen-Nissen E Smith KD Strobe KL Barrett SL Cookson BT Logan SM . Evasion of Toll-like receptor 5 by flagellated bacteria. Proc Natl Acad Sci U.S.A. (2005) 102:9247–52. doi: 10.1073/pnas.0502040102, PMID: 15956202 PMC1166605

[22] Cutler SJ Whatmore AM Commander NJ . Brucellosis–new aspects of an old disease. J Appl Microbiol. (2005) 98:1270–81. doi: 10.1111/j.1365-2672.2005.02622.x, PMID: 15916641

[23] Jiménez de Bagüés MP Terraza A Gross A Dornand J . Different responses of macrophages to smooth and rough Brucella spp.: relationship to virulence. Infect Immun. (2004) 72:2429–33. doi: 10.1128/IAI.72.4.2429-2433.2004, PMID: 15039375 PMC375206

[24] Kim S Watarai M Suzuki H Makino S Kodama T Shirahata T . Lipid raft microdomains mediate class A scavenger receptor-dependent infection of Brucella abortus. Microb Pathog. (2004) 37:11–9. doi: 10.1016/j.micpath.2004.04.002, PMID: 15194155

[25] Naroeni A Porte F . Role of cholesterol and the ganglioside GM(1) in entry and short-term survival of Brucella suis in murine macrophages. Infect Immun. (2002) 70:1640–4. doi: 10.1128/IAI.70.3.1640-1644.2002, PMID: 11854258 PMC127813

[26] Avila-Calderón ED Flores-Romo L Sharon W Donis-Maturano L Becerril-García MA Arreola MGA . Dendritic cells and Brucella spp. interaction: the sentinel host and the stealthy pathogen. Folia Microbiol (Praha). (2019) 65:1–16. doi: 10.1007/s12223-019-00691-6, PMID: 30783994 PMC7224029

[27] Fugier E Pappas G Gorvel J-P . Virulence factors in brucellosis: implications for aetiopathogenesis and treatment. Expert Rev Mol Med. (2007) 9:1–10. doi: 10.1017/S1462399407000543, PMID: 18088444

[28] Guo X Zeng H Li M Xiao Y Gu G Song Z . The mechanism of chronic intracellular infection with Brucella spp. Front Cell Infect Microbiol. (2023) 13:1129172. doi: 10.3389/fcimb.2023.1129172, PMID: 37143745 PMC10151771

[29] Martirosyan A Moreno E Gorvel J-P . An evolutionary strategy for a stealthy intracellular Brucella pathogen. Immunol Rev. (2011) 240:211–34. doi: 10.1111/j.1600-065X.2010.00982.x, PMID: 21349096

[30] Zhou Y Bu Z Qian J Chen Y Qiao L Yang S . The UTP-glucose-1-phosphate uridylyltransferase of Brucella melitensis inhibits the activation of NF-κB via regulating the bacterial type IV secretion system. Int J Biol Macromol. (2020) 164:3098–104. doi: 10.1016/j.ijbiomac.2020.08.134, PMID: 32827613

[31] Zhang J Li M Li Z Shi J Zhang Y Deng X . Deletion of the type IV secretion system effector vceA promotes autophagy and inhibits apoptosis in brucella-infected human trophoblast cells. Curr Microbiol. (2019) 76:510–9. doi: 10.1007/s00284-019-01651-6, PMID: 30805699

[32] Pizarro-Cerdá J Moreno E Sanguedolce V Mege JL Gorvel JP . Virulent Brucella abortus prevents lysosome fusion and is distributed within autophagosome-like compartments. Infect Immun. (1998) 66:2387–92. doi: 10.1128/IAI.66.5.2387-2392.1998, PMID: 9573138 PMC108212

[33] Celli J de Chastellier C Franchini D-M Pizarro-Cerda J Moreno E Gorvel JP . Brucella evades macrophage killing via VirB-dependent sustained interactions with the endoplasmic reticulum. J Exp Med. (2003) 198:545–56. doi: 10.1084/jem.20030088, PMID: 12925673 PMC2194179

[34] Ghssein G Ezzeddine Z Tokajian S Khoury CA Kobeissy H Ibrahim JN . Brucellosis: Bacteriology, pathogenesis, epidemiology and role of the metallophores in virulence: a review. Front Cell Infect Microbiol. (2025) 15:1621230. doi: 10.3389/fcimb.2025.1621230, PMID: 40697821 PMC12279742

[35] Qin Y Zhou G Jiao F Cheng C Meng C Wang L . Brucella mediates autophagy, inflammation, and apoptosis to escape host killing. Front Cell Infect Microbiol. (2024) 14:1408407. doi: 10.3389/fcimb.2024.1408407, PMID: 39507949 PMC11537862

[36] Starr T Ng TW Wehrly TD Knodler LA Celli J . Brucella intracellular replication requires trafficking through the late endosomal/lysosomal compartment. Traffic. (2008) 9:678–94. doi: 10.1111/j.1600-0854.2008.00718.x, PMID: 18266913

[37] Starr T Child R Wehrly TD . Selective subversion of autophagy complexes facilitates completion of the Brucella intracellular cycle. Cell Host Microbe. (2012) 11:33–45. doi: 10.1016/j.chom.2011.12.002, PMID: 22264511 PMC3266535

[38] Zygmunt MS Blasco JM Letesson J-J Cloeckaert A Moriyón I . DNA polymorphism analysis of Brucella lipopolysaccharide genes reveals marked differences in O-polysaccharide biosynthetic genes between smooth and rough Brucella species and novel species-specific markers. BMC Microbiol. (2009) 9:92. doi: 10.1186/1471-2180-9-92, PMID: 19439075 PMC2698832

[39] Bao Y Tian M Li P Liu J Ding C Yu S . Characterization of Brucella abortus mutant strain Δ22915, a potential vaccine candidate. Vet Res. (2017) 48:17. doi: 10.1186/s13567-017-0422-9, PMID: 28376905 PMC5381064

[40] Dong B Li F Wang J Lv S Miao L Guo G . Effect of ubiquitin-proteasome system and autophagy-lysosome pathway on intracellular replication of Brucella.suis. Vet Microbiol. (2023) 280:109699. doi: 10.1016/j.vetmic.2023.109699, PMID: 36812863

[41] Zhu J Dong Q Dong C Zhang X Zhang H Chen Z . Global lysine crotonylation alterations of host cell proteins caused by brucella effector bspF. Front Cell Infect Microbiol. (2021) 10:603457. doi: 10.3389/fcimb.2020.603457, PMID: 33489935 PMC7821425

[42] Pellegrini JM González-Espinoza G Shayan RR Hysenaj L Rouma T Arce-Gorvel V . Brucella abortus impairs T lymphocyte responsiveness by mobilizing IL-1RA-secreting omental neutrophils. Nat Commun. (2025) 16:862. doi: 10.1038/s41467-024-55799-2, PMID: 39833171 PMC11747348

[43] Dadelahi AS Abushahba MFN Ponzilacqua-Silva B Chambers CA Moley CR Lacey CA . Interactions between B cells and T follicular regulatory cells enhance susceptibility to Brucella infection independent of the anti-Brucella humoral response. PloS Pathog. (2023) 19:e1011672. doi: 10.1371/journal.ppat.1011672, PMID: 37721965 PMC10538787

[44] Li R Liu W Yin X Zheng F Wang Z Wu X . Brucella spp. Omp25 Promotes Proteasome-Mediated cGAS Degradation to Attenuate IFN-β Production. Front Microbiol. (2021) 12:702881. doi: 10.3389/fmicb.2021.702881, PMID: 34394047 PMC8358459

[45] Harms JS Khan M Hall C Splitter GA Homan EJ Bremel RD . Brucella peptide cross-reactive major histocompatibility complex class I presentation activates SIINFEKL-specific T cell receptor-expressing T cells. Infect Immun. (2018) 86:e00281–18. doi: 10.1128/IAI.00281-18, PMID: 29735518 PMC6013681

[46] Serafino A Bertinat YA Bueno J Pittaluga JR Birnberg Weiss F Milillo MA . Beyond its preferential niche: Brucella abortus RNA down-modulates the IFN-γ-induced MHC-I expression in epithelial and endothelial cells. PloS One. (2024) 19:e0306429. doi: 10.1371/journal.pone.0306429, PMID: 38980867 PMC11232970

[47] Velásquez LN Milillo MA Delpino MV Trotta A Mercogliano MF Pozner RG . Inhibition of MHC-I by Brucella abortus is an early event during infection and involves EGFR pathway. Immunol Cell Biol. (2016) 95:388–98. doi: 10.1038/icb.2016.111, PMID: 27811842

[48] Su X Zhao S Song Y . Expression of NLRP3 and AIM2 inflammasome in Peripheral blood in Chinese patients with acute and chronic brucellosis. Sci Rep. (2022) 12:15123. doi: 10.1038/s41598-022-19398-9, PMID: 36068262 PMC9448728

[49] Hu H Tian M Yin Y Zuo D Guan X Ding C . Brucella induces heme oxygenase-1 expression to promote its infection. Transbound Emerg Dis. (2021) 69:2697–711. doi: 10.1111/tbed.14422, PMID: 34918880

[50] Canning PC Roth JA Tabatabai LB Deyoe BL . Isolation of components of Brucella abortus responsible for inhibition of function in bovine neutrophils. J Infect Dis. (1985) 152:913–21. doi: 10.1093/infdis/152.5.913, PMID: 2995513

[51] Bouhet S Lafont V Billard E Gross A Dornand J . The IFNgamma-induced STAT1-CBP/P300 association, required for a normal response to the cytokine, is disrupted in Brucella-infected macrophages. Microb Pathog. (2008) 46:88–97. doi: 10.1016/j.micpath.2008.10.011, PMID: 19041714

[52] Wang Z Bie P Cheng J Lu L Cui B Wu Q . The ABC transporter YejABEF is required for resistance to antimicrobial peptides and the virulence of Brucella melitensis. Sci Rep. (2016) 6:31876. doi: 10.1038/srep31876, PMID: 27550726 PMC4994006

[53] Xu Z Tong Z Zhang H Deng X Yang N Wang Z . Effects of major OMPs and LPS of Brucella on the control of activation of bone marrow-derived dendritic cells and proliferation of T-lymphocytes in mice. Iran J Vet Res. (2024) 25:224–32. doi: 10.22099/ijvr.2024.48969.7177, PMID: 39925832 PMC11801326

[54] Billard E Dornand J Gross A . Brucella suis prevents human dendritic cell maturation and antigen presentation through regulation of tumor necrosis factor alpha secretion. Infect Immun. (2007) 75:4980–9. doi: 10.1128/IAI.00637-07, PMID: 17635859 PMC2044515

[55] Jiang S Ma J Li Y Lu B Du J Xu J . A polysaccharide from native Curcuma kwangsiensis and its mechanism of reversing MDSC-induced suppressive function. Carbohydr Polym. (2022) 297:120020. doi: 10.1016/j.carbpol.2022.120020, PMID: 36184172

[56] Wang Y Yang S Han B Du X Sun H Du Y . Single-cell landscape revealed immune characteristics associated with disease phases in brucellosis patients. Imeta. (2024) 3:e226. doi: 10.1002/imt2.226, PMID: 39135683 PMC11316929

[57] Zhang G Shen Q Ye J Feng Y Boireau P Fan X . Single-cell transcriptome profiling reveals the immune dysregulation characteristics of mice infected by Brucella abortus. J Infect Dis. (2025) 11:jiaf522. doi: 10.1093/infdis/jiaf522, PMID: 41074555 PMC12811882

[58] Xie D Xu H Su C Lu J Shen W Li P . Brucella infection induces chromatin restructuring in host cells to activate immune responses. Front Immunol. (2025) 16:1574006. doi: 10.3389/fimmu.2025.1574006, PMID: 40539063 PMC12176892

